# Weight-Bearing CT for Diseases around the Ankle Joint

**DOI:** 10.3390/diagnostics14151641

**Published:** 2024-07-30

**Authors:** Jahyung Kim, Jaeyoung Kim, Saintpee Kim, Young Yi

**Affiliations:** 1 Department of Orthopedic Surgery, Seoul National University Hospital, Seoul National University College of Medicine, Seoul 03080, Republic of Korea; hpsyndrome@naver.com; 2 Baylor University Medical Center, Dallas, TX 75246, USA; jaeyoungkimj@gmail.com; 3 Department of Orthopedic Surgery, Gangbuk Etteum Hospital, Seoul 01170, Republic of Korea; ksb8060@gmail.com; 4 Department of Orthopedic Surgery, Sanggye Paik Hospital, Inje University College of Medicine, Seoul 01757, Republic of Korea; 5 Department of Orthopaedic Surgery and Rehabilitation, Yale School of Medicine, New Haven, CT 06510, USA

**Keywords:** weight-bearing, CT, ankle arthritis, syndesmosis injury, osteochondral lesion of talus, ankle instability

## Abstract

Weight-bearing computed tomography (WBCT) enables acquisition of three-dimensional bony structure images in a physiological weight-bearing position, which is fundamental in understanding the pathologic lesions and deformities of the ankle joint. Over the past decade, researchers have focused on validating and developing WBCT measurements, which has significantly enhanced our knowledge of common foot and ankle diseases. Consequently, understanding the application of WBCT in clinical practice is becoming more important to produce improved outcomes in the treatment of disease around the ankle joint. This review will describe an overview of what is currently being evaluated in foot and ankle surgery using WBCT and where the course of research will be heading in the future.

## 1. Introduction

Understanding the foot and ankle region is challenging for orthopedic surgeons due to its complex anatomical and biomechanical interrelations. In addition, it is essential to appreciate their altered position and alignment with weight-bearing to fully address a patient’s symptoms during walking or running. In other words, pathologic conditions such as impingement, joint space narrowing, and malalignment may only be apparent with load during assessment, which could be left undiagnosed if evaluated without weight-bearing [1].

Although conventional standing radiographs allow clinicians to identify the ankle in a weight-bearing position, these provide limited information to foot and ankle surgeons owing to their low spatial resolution and overlaps with adjacent bones [2]. To overcome the shortcomings of computed tomography (CT) and magnetic resonance imaging (MRI), which are usually obtained in a supine manner, several attempts have been made to evaluate foot and ankle pathologies under simulated weight-bearing conditions using custom-made loading devices [3,4]. Unfortunately, these devices only enable partial weight-bearing, which could underestimate the physiologic loading of the ankle joint compared with normal fully-weight-bearing positions. In addition, such methods could not reflect muscle activation, which plays a significant role in determining the positions of the bones and their relationships with the joints.

Cone-beam CT (CBCT), which has been primarily utilized in dental diagnostics, involves the use of a cone-shaped X-ray beam and two-dimensional (2D) detectors that acquire volumetric data with less rotation of the X-ray source compared with conventional CT [2]. Because the detector moves around the patient during CBCT scanning, development of weight-bearing CT (WBCT) became possible and its adoption in the foot and ankle area permitted an important technical step forward. It enables acquisition of bony structure images in a physiological weight-bearing position, which is fundamental to apprehending the degenerative lesions and deformities of the ankle joint. In practice, weight-bearing CBCT combines the advantages of high-spatial-resolution three-dimensional (3D) imaging with weight-bearing and muscle activation, allowing the exact reproduction of dimensions and proportions. Additional advantages of WBCT include a rapid image acquisition time, a reduced cost, and a low radiation dose [5].

Over more than ten years of its usage in foot and ankle surgery, numerus studies have been executed to better utilize WBCT in clinical practice. Researchers have focused on developing and validating the WBCT measurements and providing information that cannot be obtained in conventional methods. Such efforts have significantly enhanced our knowledge of foot and ankle pathologic conditions. The purpose of this review article is to introduce radiologic measurements of WBCT and their ongoing clinical usage in diseases around the ankle joint: osteoarthritis, syndesmotic injury, osteochondral lesion of talus, chronic ankle instability, and acute ankle sprain.

## 2. Normal Anatomy

Since its introduction in foot and ankle surgery, WBCT has been used to study the normal anatomy of the foot and ankle in patients without significant pathology. In particular, WBCT has been frequently applied to healthy control patients to discover rotational movements of the talus within the mortise and subtalar joint configuration, which could not be evaluated through weight-bearing plain radiograph due to superimposition of bones [6,7]. Lepojärvi et al. investigated the talar movement by measuring the rotation of the talus, medial clear space, anterior and posterior widths of the tibiotalar joint, transition of the talus, and talar tilt in 32 healthy subjects [6]. They concluded that when the ankle is taken from maximal internal to maximal external rotation, the talus rotates a total of 10 degrees internally–externally about the tibia with no substantial widening of the medial clear space. In order to determine the morphology and orientation of the posterior facet of the subtalar joint, Collin et al. analyzed 59 healthy volunteers without hindfoot and ankle pathologies [7]. They found that the posterior facet was concave in 88% of subjects and flat in 12%, while it was oriented valgus in 90% and varus in 10% when measured in the middle coronal plane. The authors added that the posterior facet of the subtalar joint is consistently oriented in valgus, whereas the most anterior aspect of the posterior facet of the subtalar joint is typically oriented in varus. This study suggests that the measurements of the varus–valgus orientation of the posterior facet of the subtalar joint could be dependent on where the CT image is taken in the anterior–posterior direction.

Another study by Ritcher et al. combined WBCT with a custom pedography sensor to describe the relative position of the anatomic foot center and the pedographic center of gravity during weight-bearing. The authors analyzed 180 feet in 90 patients and found that the anatomic foot center was distal to the center of gravity of the foot in 97% of feet at a mean distance of 27.5 mm, while it was lateral to the center of gravity in 62% of feet, by a mean of 2.0 mm. This finding suggests that there is a constant major distal longitudinal shift of center of gravity relative to foot center and an inconstant minor mediolateral shift [8].

## 3. Diseases around the Ankle Joint

### 3.1. Ankle Osteoarthritis

WBCT offers significant clinical advantages in ankle osteoarthritis (OA) because thinning of the cartilage and manifestation of deformities around the ankle joint become more evident under load [9]. It also better reflects geometric characteristics of the ankle joint and allows for more accurate measurements compared with a conventional radiograph [2]. WBCT is therefore utilized for precise diagnosis and classification of the pathologic condition, along with detailed establishment of therapeutic strategies.

#### 3.1.1. Diagnosis

**Figure 1:**
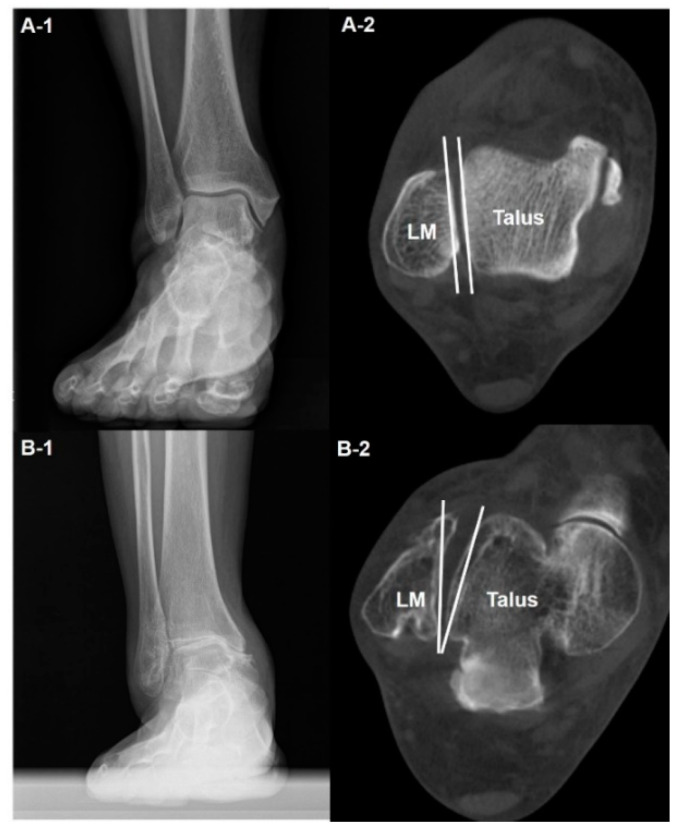
Abnormal internal rotation of talus in the axial plane in varus ankle arthritis. Plain weight-bearing anteroposterior ankle radiography of the normal ankle (**A-1**) with corresponding WBCT axial image showing the congruent position of the talus in the ankle joint, without rotation against the lateral malleolus (LM) (**A-2**). Plain weight-bearing anteroposterior ankle radiography of a patient with varus ankle osteoarthritis (**B-1**), with WBCT axial image showing the internally rotated talus against the lateral malleolus (LM) (**B-2**).

The use of WBCT in the diagnosis of ankle OA begins with a stereotaxic understanding of the position of the talus relative to the ankle mortise. The amount of pathologically altered ankle structure may suggest severity of ankle arthritis. Kim et al. used the talus rotation ratio to quantify abnormal internal rotation of the talus and concluded that the talus is abnormally internally rotated in patients with varus ankle arthritis [10]. They also added that patients with severe varus ankle osteoarthritis showed a higher incidence of abnormal talus internal rotation compared with those with moderate varus ankle osteoarthritis (Figure 1). Song et al. evaluated the amount of correction after SMO using an axial loading CT, which showed significantly corrected abnormal internal rotation of the talus [4]. These findings indicate that not only the coronal and sagittal pathologic components of the ankle OA that can easily be visualized with conventional radiographs, but also the axial component that can only be obtained with WBCT should be considered.

**Figure 2:**
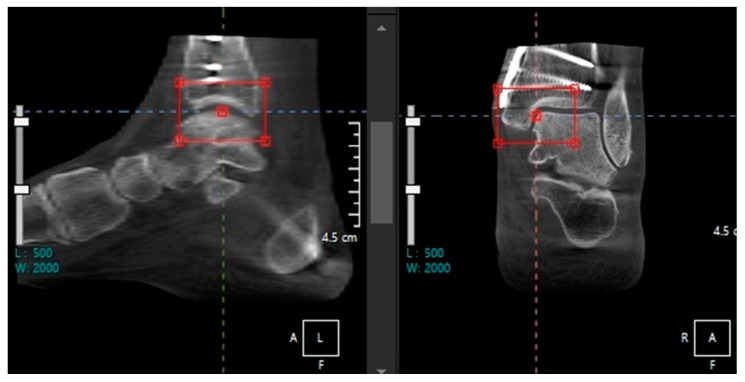
Bounding box method to detect the joint width narrowing using automated intelligence (AI) with the Xelis 3D imaging software ver 1.0 (INFINITT Healthcare, Seoul, Korea).

In addition, several studies approached diagnosis of ankle arthritis by measuring the joint space width (JSW) using WBCT. Wiley et al. first applied JSW to evaluate the joint space narrowing in posttraumatic ankle osteoarthritis, which showed good reliability and reproducibility [11]. The measurement was performed in a single-value, 2D manner made at selected locations. With the development of technology, however, software-driven 3D geometric measurements of the distance between bone surfaces became possible (Figure 2) [12]. Although its automated imaging process of the measurements needs to be improved, 3D JSW mapping may enhance intuitive visualization of ankle joint biomechanics. Furthermore, it provides highly sensitive monitoring of joint space narrowing, which may be used as an alternative indicator for progression in joint disease.

**Figure 3:**
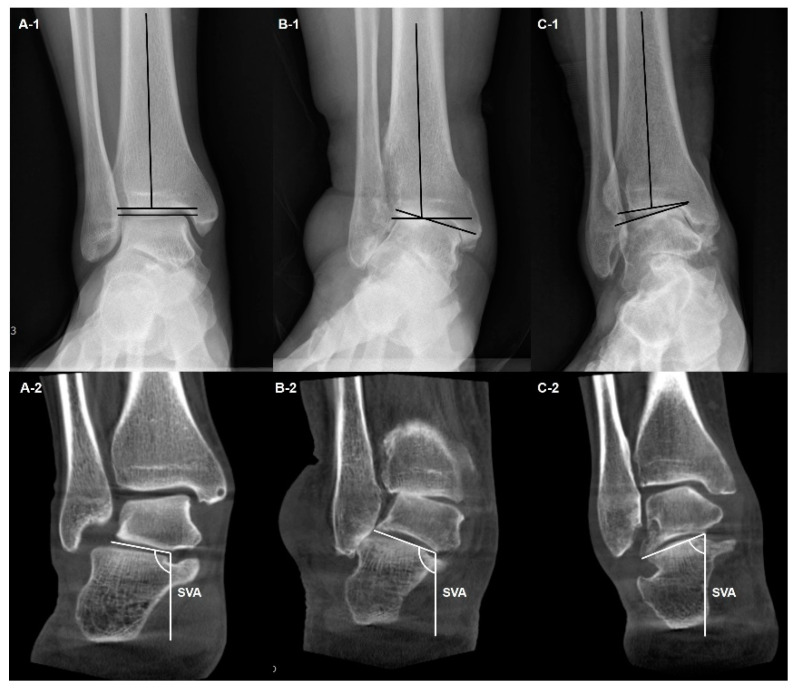
Weight-bearing plain radiographs of patients with various types of tibiotalar alignment (**A-1**,**B-1**,**C-1**) and corresponding subtalar joint orientation in WBCT (**A-2**,**B-2**,**C-2**). The subtalar vertical angle (SVA) was defined as the inclination of the line connecting the medial and lateral aspects of the talus with a vertical line, which is perpendicular to the ground.

The hindfoot alignment and the compensation of the subtalar joint should also be taken into consideration when making therapeutic strategies for ankle OA. Because the measurements in hindfoot alignment view are known to be sensitive to changes in X-ray beam projection angle, WBCT is particularly useful for evaluating hindfoot alignment [13,14]. Krähenbühl et al. evaluated the subtalar joint orientation using coronal WBCT images and concluded that the subtalar joint was more varus oriented in varus ankle arthritis while more valgus oriented in valgus ankle arthritis (Figure 3) [15]. They also studied the degree of inframalleolar compensation against supramalleolar abnormalities with WBCT, stating that subtalar joint compensation occurred in varus ankle arthritis whereas it did not happen in valgus ankle arthritis. Similarly, Kang et al. focused on subtalar compensation in advanced varus ankle arthritis and found that ankle OA with talar tilt angle greater than 9.5 degrees is significantly prone to a non-compensated heel [16]. Likewise, WBCT is actively being used to explain the hindfoot alignment and subtalar joint characteristics in ankle OA.

#### 3.1.2. Classification

The Takakura classification is based on the ankle mortise weight-bearing plain radiograph findings, which is useful when monitoring the progression of arthritis [17]. Although it is probably the most widely used classification worldwide, there are some forms of arthritis that cannot be explained by this system because it solely uses a 2D coronal plane image. Kim et al. measured the ratio of medial gutter width to tibial plafond–talar dome space in coronal images of the anterior, middle, and posterior ankle, defining medial gutter narrowing as a ratio of less than 0.5 [18]. Interestingly, the anterior part of the ankle joint showed valgus talar tilt rather than varus, contradicting our existing belief that medial gutter arthritis is a feature of a subtype of varus ankle arthritis described in the Takakura classification. Other pain radiograph-based classifications may also be limited in wholly reflecting the 3D characteristics of ankle OA [19,20].

**Figure 4:**
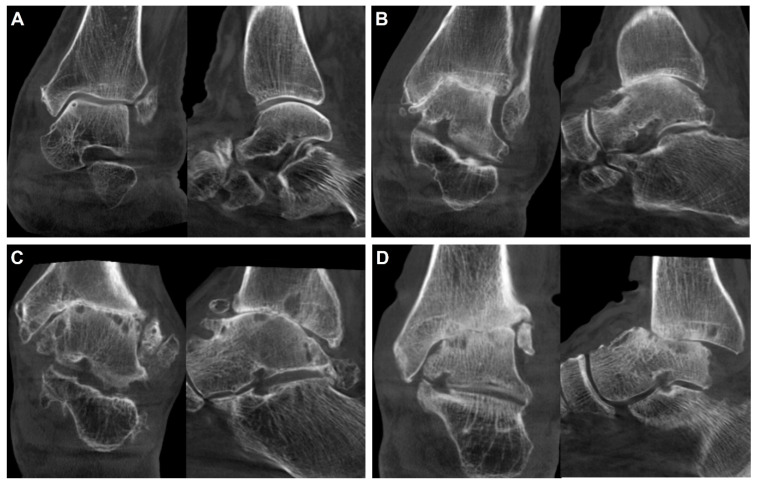
A classification system proposed by Richter et al. [21]. (**A**) First degree of osteoarthritis with osteophyte formation and joint space narrowing, but not complete loss. (**B**) Second degree of osteoarthritis with partial or complete loss of joint space. (**C**) Third degree of osteoarthritis with additional subchondral cysts, with remaining joint surface congruency. (**D**) Fourth degree of osteoarthritis with aggravated joint surface destruction and incongruence.

Using WBCT, Richter et al. proposed a classification, which subdivided ankle OA into four degrees [21]. The first-degree ankle OA includes joint space narrowing, which is not a complete loss, and osteophyte formation (Figure 4A). The second degree includes a partial or total loss of joint space (Figure 4B). The third degree implies the presence of additional subchondral cysts with remaining joint surface congruence (Figure 4C). The fourth degree includes additional joint destruction with incongruity of the articular surface (Figure 4D). The authors commented that the new classification may effectively combine the classic features of osteoarthritis (joint space narrowing, osteophyte formation, and subchondral cysts) along with 3D visualization.

In addition, Tazegul et al. developed and described a computational method that enables objective quantitative assessment of OA using WBCT image intensity, i.e., Hounsfield units [9]. The authors used four projections at different locations throughout the joint and allowed intuitive visualization in which quadrants have reduced joint space width and contrast. Although the study simply proposed a computational methodology with small sample size, their findings suggest that quantitative measurement and appropriate selection of objective points within each joint may further improve the reliability and reproducibility of OA classification.

#### 3.1.3. Treatment

##### Deformity Correction Surgery

**Figure 5:**
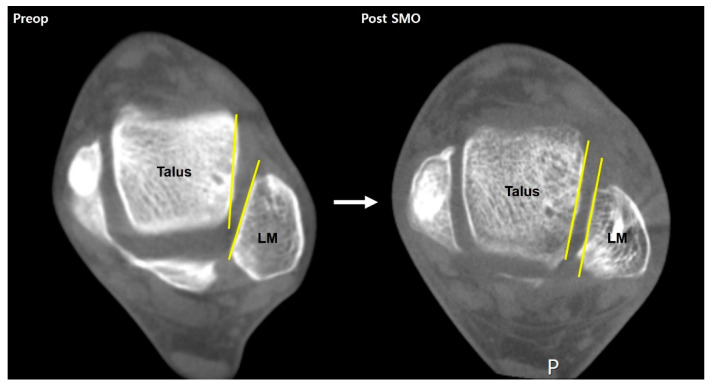
A case of a varus ankle osteoarthritis patient showing correction of abnormal internal rotation of the talus after supramalleolar osteotomy (SMO).

Supramalleolar osteotomy (SMO) is an established surgical option to correct valgus or varus deformity in patients presenting with ankle OA [22]. It is a joint preserving modality that is utilized for management of early- to mid-stage asymmetric ankle arthritis [23]. The procedure may bring about load shift of the weight-bearing axis, thereby redistributing and decreasing the peak stress concentration within the ankle mortise [24]. WBCT is being used to preoperatively determine the expected amount of correction and to postoperatively evaluate the changes in ankle joint position in a 3D manner (Figure 5) [4]. Furthermore, Burssens et al. described changes in subtalar joint alignment in sagittal and axial planes after SMO [25]. Their findings indicate that WBCT can be used to describe subtalar joint behavior in ankle arthritis that might be ignored in 2D approaches.

WBCT can also improve the accuracy of corrective osteotomies by assisting development of a patient-specific guide. Using the patient-specific guide, the appropriate level of the osteotomy can be determined based on the fit of the guide and a virtual correction can be performed preoperatively until the desired position is achieved [21]. In their pilot study, Facit et al. suggested that a dome-shaped supramalleolar osteotomy using 3D-printing guides designed on WBCT can be used to potentially mitigate the technical drawbacks of free-hand osteotomies [26].

##### Ankle Replacement Surgery

Total ankle arthroplasty (TAA) has emerged as a viable option for end-stage ankle OA [27]. It has gained significant popularity within the last decades among foot and ankle surgeons with drastically increasing usage rates [28,29,30,31]. Such an increasing trend for using this procedure might be due to some of its advantages compared with ankle arthrodesis: the ability to preserve ankle joint motion, to provide a more physiologic gait, and to minimize the risk of adjacent joint arthritis [32,33,34]. With technical improvements in implant design and surgical technique, multiple studies have proven that TAA can provide equivocal or better clinical outcomes than ankle arthrodesis [35,36].

In order to produce satisfactory outcomes and minimize failure, implants must be properly placed within the ankle joint [37]. In this sense, WBCT-based patient-specific instrumentation guides make accurate bony resection based on patient-specific anatomy possible in TAA. Because there is no intraoperative navigation system for TAA currently available, integrating WBCT into the preoperative planning process enables determination of potential implant size and positions, which would allow for less variability in the operating room [38].

Preoperative WBCT may also be utilized in determining the adjunctive procedures to be performed along with TAA. de Cesar Netto et al. utilized preoperative WBCT-generated foot and ankle offset (FAO) to retrospectively review the number of corrective alignment bony procedures performed at the time of TAA [39]. They concluded that the number of additional osseous realignment procedures significantly correlated with preoperative FAO, with valgus malalignment patients requiring a greater number compared with varus patients. The findings can be implied that preoperative FAO can predict the number of bony realignment procedures necessary for TAA, which may eventually enhance the preoperative assessment and surgical planning for patients undergoing TAA.

Although TAA is now indicated as an appropriate treatment modality for end stage ankle arthritis, complications following joint replacement surgery are still reported in the literature with considerable incidence rates. A recent systematic analysis reviewed 22 studies which included 4412 ankles from 4276 patients who underwent TAA, with a mean follow-up of 66.6 ± 40.9 months [40]. The review reported an adjusted mean complication rate of 23.7% (2.4–52%), mostly of high-grade complications (35.6%) such as deep infection, aseptic loosening, or implant failure [41].

**Figure 6:**
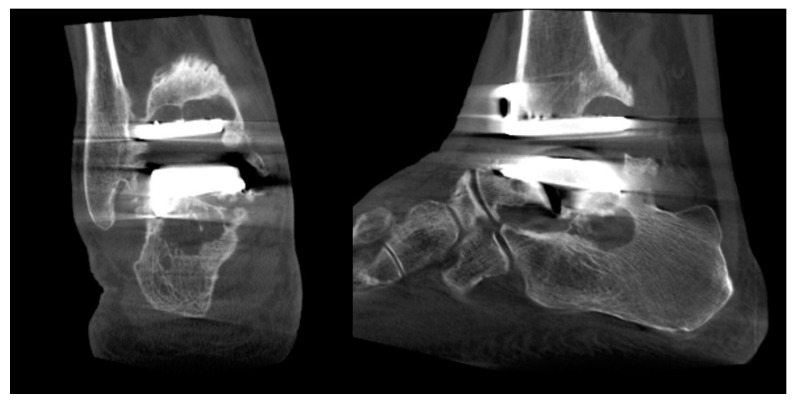
Multiple periprosthetic cysts can be detected using WBCT.

The most common complications known to contribute to revision surgery after TAA are deep infection, aseptic loosening, and subsidence [33,42]. In this regard, WBCT can be an effective postoperative diagnostic option to detect early implant failure or periprosthetic cyst formation (Figure 6). Lintz et al. found out that 81% of 60 patients who underwent TAA had periprosthetic cysts in WBCT at a mean follow-up of 44.6 months [43]. They also demonstrated threshold values of FAO for patients with residual malalignment following TAR, where values below −2.75% in varus cases and above 4.5% FAO in valgus cases could predict increased risks of periprosthetic cyst formation. In light of these findings, it seems reasonable to recommend WBCT scans for complete 3D assessment of the alignment and early detection of complications in patients that have undergone TAR.

### 3.2. Syndesmosis Injury

Untreated or inadequately treated syndesmosis injuries can lead to significant long-term issues such as pain, instability, and degenerative changes in the ankle joint [44]. Unfortunately, precise diagnosis of syndesmotic injuries remains difficult, especially if the injury is subtle. In fact, diagnosis of syndesmosis injury has relied on various imaging modalities, which have inherent limitations. First, the inability of conventional CT or MRI to evaluate the syndesmotic structures under load or stress limits their reliability. Secondly, establishing the normal range indicating abnormal radiographic measurement in syndesmosis injury is challenging because of significant anatomic variation among individuals. For this reason, experts recommend using the unaffected contralateral ankle as a reference to determine the abnormality of the syndesmosis [45,46].

WBCT is being acknowledged as a useful diagnostic tool owing to its ability to directly assess the biomechanics of the tibiofibular joint under physiologic load. In addition, simultaneous visualization of the contralateral ankle under equivalent physiologic load makes WBCT imaging more beneficial in evaluating syndesmotic injuries. In accordance with such advantages, several studies have focused on diagnostic applications of WBCT in syndesmotic injuries [47].

**Figure 7:**
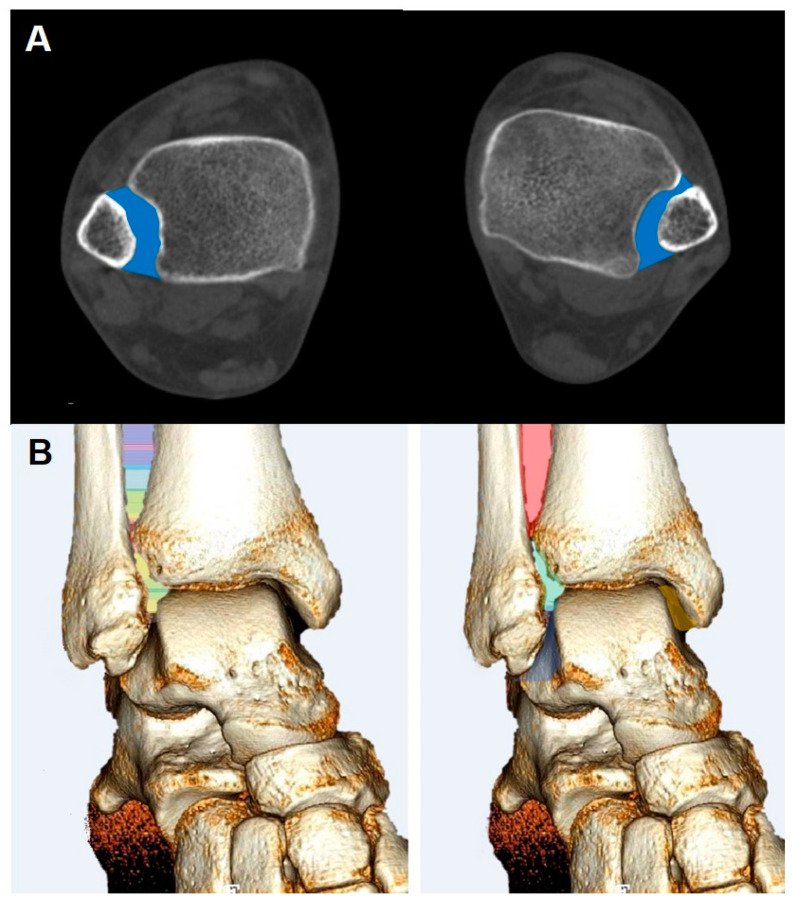
Methods to estimate the syndesmosis injury. (**A**) Circumferential area measurement. (**B**) Volumetric measurement.

Recently, instead of relying on linear or angular measurements, multiples studies have introduced dimensional or volumetric assessment using WBCT, which has been shown to be accurate in estimating syndesmotic injury. Hagemeijer et al. described circumferential area measurement using WBCT and found that patients with unilateral syndesmotic instability showed a significantly increased syndesmotic area of the injured side compared with the contralateral uninjured side [48]. To better induce syndesmotic widening, studies have demonstrated the addition of external rotation torque during WBCT measurements and confirmed better diagnostic accuracy [49,50]. In this regard, Shamrock et al. reported normal threshold values of syndesmotic area measurements [51]. In over 50 uninjured ankles that underwent WBCT scans with external rotation stress, axial–plane WBCT images measured 1 cm proximal to the apex of the tibial plafond showed a mean syndesmotic area of 106.7 ± 18.3 mm^2^. Beyond this 2D area measurement, advanced measurement modalities like volumetric measurement would allow for improved diagnostic efficiency for the syndesmotic area in the future (Figure 7) [52].

### 3.3. Osteochondral Lesion of Talus

**Figure 8:**
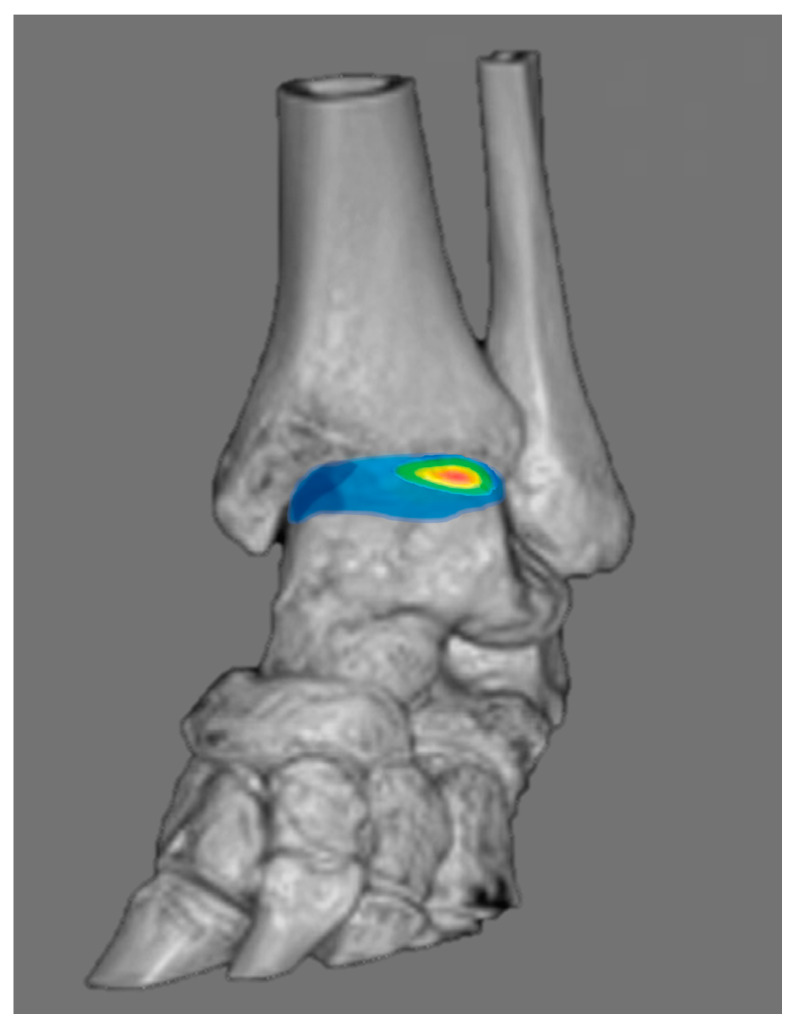
Illustration showing distance mapping analysis using Xelis 3D imaging software ver 1.0 (INFINITT Healthcare, Seoul, Korea).

The keys to successful therapeutic outcomes for osteochondral lesion of talus (OLT) may depend on the surgeon’s ability to optimize adequate surgical intervention to the lesion. As a result, it is critical to precisely measure the lesion’s surface, volume, and depth during preoperative planning in OLT surgery. Conventional imaging modalities like CT or MRI, however, are known to over- or underestimate the lesion’s measurements [53,54]. Alternatively, distance mapping (DM), which is a new imaging modality that provides quantitative visualization of the joint surface distance distribution on each articular bone, may offer some possibilities for more accurate evaluation of the lesion’s dimension in OLT when used with WBCT (Figure 8) [55,56]. In other words, intuitive assessment of the OLT is possible because DM provides information regarding distance between bones via color-coded maps.

Efrima et al. measured the surface, depth, and volume of 40 OLTs using WBCT and DM and estimated the reliability of a renewed imaging modality [57]. The interclass correlation of the measurements using the devices showed excellent inter-rater and intra-rater agreement. Subsequently, when asked to choose the therapeutic options based on the measurement, evaluators achieved a near-perfect agreement on the preferred surgical approach. Although further studies would be needed to support their findings, the introduction of WBCT along with DM in OLT surgery may hold significant implications for enhancing agreement for preoperative planning.

### 3.4. Chronic Ankle Instability

Compared with other disease entities around the ankle joint, the literature regarding the use of WBCT in chronic ankle instability (CAI) is limited. Previous studies have focused on correlation between bony alignment and CAI. Van Bergeyk et al. compared the hindfoot alignment of 12 patients with CAI with 12 control patients using simulated WBCT and found that measurements indicating calcaneal varus deformity showed 3 to 4 degrees more varus in the CAI patients than the control patients [58]. The researchers concluded that hindfoot varus alignment was a significant risk factor contributing to recurrent ankle instability. Lintz et al. confirmed the finding in their comparative study of 34 patients with CAI and 155 patients without ankle instability [59]. They found a 35% increased odds ratio of CAI per 1% increase in varus hindfoot alignment.

**Figure 9:**
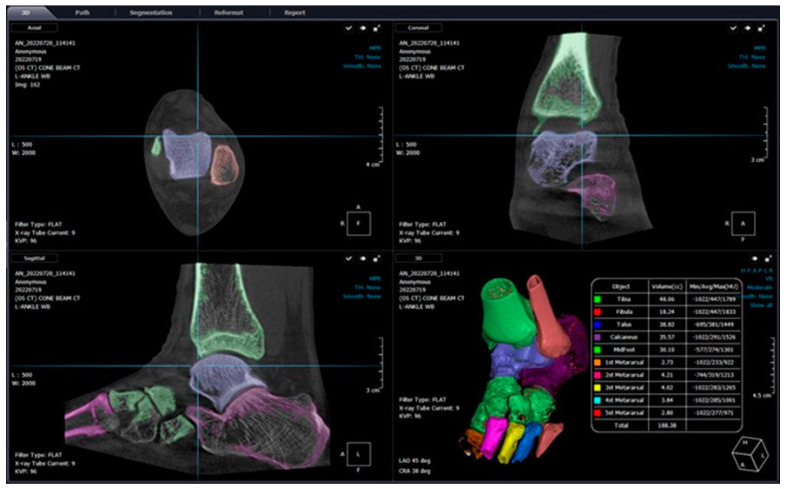
Semi-automatic segmentation of the bones around the ankle joint using Xelis 3D imaging software ver 1.0 (INFINITT Healthcare, Seoul Korea).

In their narrative review, Lintz et al. proposed possible usages of dynamic WBCT imaging to better explore CAI [60]. They described that traditional varus and anterior drawer dynamic imaging of the bilateral ankle is possible with WBCT, which may provide vast amounts of information that cannot be obtained from conventional dynamic radiographs. First, automatic axes calculation or simultaneous associated fracture detection can be possible with semiautomatic segmentation (Figure 9). Second, with the use of DM, objective assessment of tibiotalar tilt would be available because it offers an intuitive colorized map along with precise numeric quantification of the distance between articular surfaces in a defined area. Finally, utilization of supplementary devices like customized jigs to provoke stress can minimize some of the downsides of dynamic imaging: poor reproducibility and increased motion artifacts.

### 3.5. Acute Ankle Sprain

Following acute ankle sprain, multiple views, such as oblique or lateral views, are often required in a conventional radiographic setup to detect lesions that are not clearly visible owing to bone superimposition. Furthermore, there is a belief that radiographs in acute ankle sprain should not be performed in a standing manner because weight-bearing would provoke pain or deformation of the injured limb in the context of trauma. Consequently, additional conventional CT is often administered for patients with suspected bony lesions, which will eventually result in high radiation exposure and time consumption for patients.

**Figure 10:**
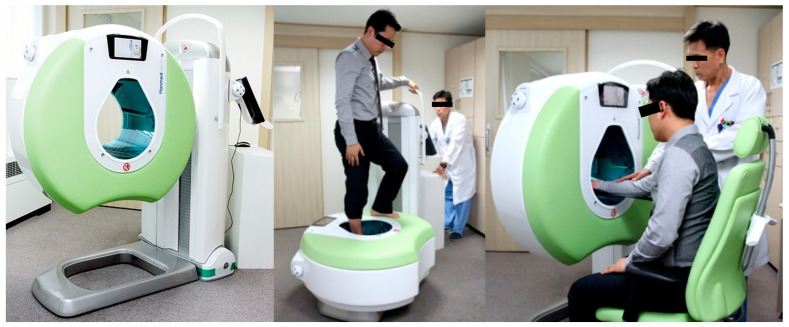
Most of the commercially available WBCTs provide a mobile device to enable patients to remain seated during the examination (Planmed Verity Extremity, Planmed Oy, Helsinki, Finland).

**Figure 11:**
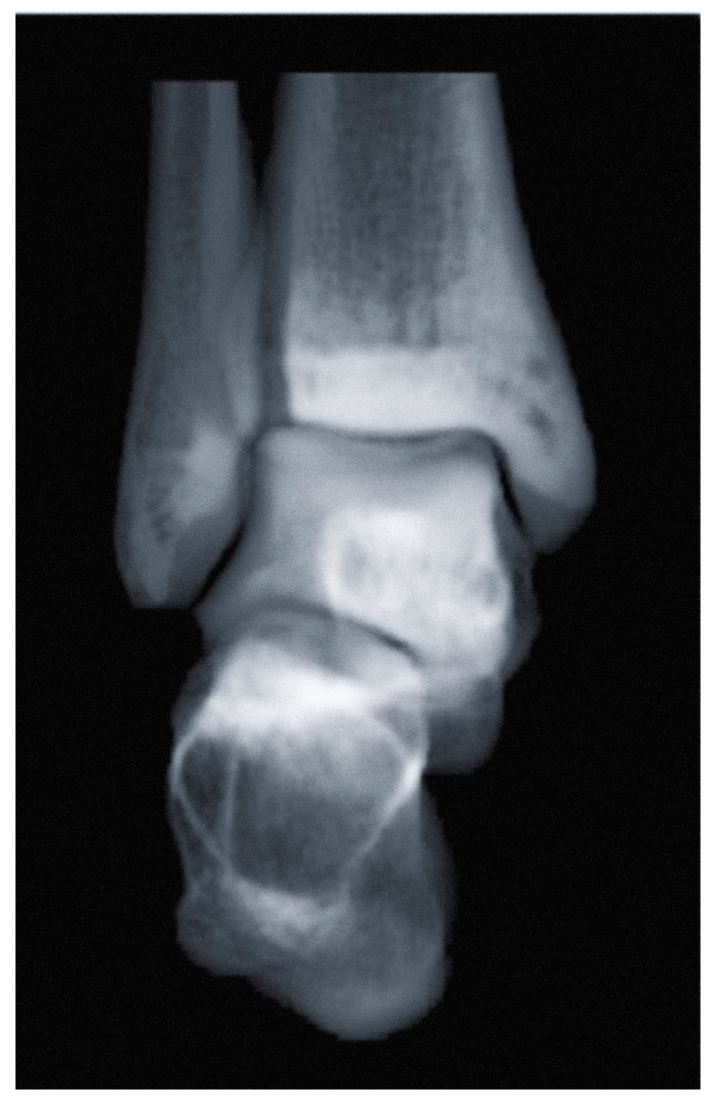
A digitally reconstructed radiograph (DRR) anteroposterior view of the ankle joint.

In fact, WBCT does not have to be taken with the patient standing. While the term “weight-bearing” was applied because of its historical appeal to surgeons, most available products provide a mobile device that enables patients to remain seated during the examination (Figure 10). Moreover, digitally reconstructed radiographs (DRR) can now be created and implemented using WBCT (Figure 11) [61]. As a result, surgeons confronting acute ankle sprains may benefit from an all-in-one radiographic modality that could offer delicate spatial resolution of 0.27 to 0.35 mm for enhanced diagnosis, without causing pain or discomfort to injured patients. Jacques et al. executed a prospective comparative study on 165 emergency patients who underwent the classical 2D X-ray-multi-detector CT pathway versus 224 patients for who CBCT was used as a primary imaging modality [62]. They reported a 35% reduction in radiation dose and a 15% reduction in turnover time. In summary, WBCT can effectively be used in acute ankle sprain to reduce the number of false-negatives or delayed diagnoses of concomitant pathology, radiation exposure, time to diagnosis, time spent in the emergency department, and direct and secondary costs [60].

## 4. Conclusions

Over the course of a decade, WBCT has changed the understanding of pathologic conditions around the ankle joint and is currently being used to revolutionize therapeutic consequences for foot and ankle surgeons. Moreover, there is still a vast opportunity for future research with WBCT to minimize redundant preoperative diagnostic processes and maximize postoperative outcomes. In fact, WBCT is yet to be globally used because there is much to be improved, such as limitations in automatic measurement or the necessity of specialized software or professionals for 3D analysis [63,64]. Nevertheless, with technological improvement, we believe that WBCT will be established as a standard diagnostic imaging tool for disorders of the ankle joint.
